# Fundoplication significantly improves objective and subjective reflux outcomes—a meta-analysis

**DOI:** 10.1007/s00464-025-11856-5

**Published:** 2025-05-29

**Authors:** Yanick Tadé, Daniel Newman, Ryan W. Walters, Kalyana C. Nandipati

**Affiliations:** 1 School of Medicine, Creighton University, Omaha, NE USA; 2 Department of Clinical Research and Public Health, Creighton University, Omaha, NE USA; 3 Department of Surgery, Atrium Health, Wake Forest School of Medicine, 2630 E. 7 th St., Ste 100, Charlotte, NC 28204 USA

**Keywords:** GERD, MSA, Fundoplication, Meta-analysis, LNF, Systematic review

## Abstract

**Introduction:**

Gastroesophageal reflux disease (GERD) impacts 10–30% of the population in the Western world. Surgical interventions including Laparoscopic Fundoplication (LF), Transoral Incisionless Fundoplication (TIF), and Magnetic Sphincter Augmentation (MSA) have proven effective in managing GERD. This meta-analysis aims to compare short- and long-term outcomes of these surgical options.

**Methods:**

A comprehensive search of PubMed, Embase, Scopus, Cochrane, and Medline from 1980 to 2024 was conducted to identify randomized control trials or cohort designs that included adults with GERD who underwent fundoplication (Nissen, Toupet), MSA, or TIF and had preoperative and post-operative acid exposure time (AET), DeMeester score, and/or relief score (e.g., GERD-HRQL); we considered all post-operative outcome measurements. We excluded studies with any surgical variations of the procedures, reoperations, and studies not published in English. Risk of bias was assessed using the Oxford scoring system for randomized control trials and the Newcastle–Ottawa scale (NOS) for cohort designs. A total of 3912 studies were identified initially, with our review including 78 unique studies providing 166 post-operative outcome measurements. Given studies could include multiple post-operative outcome measurements, we used multilevel random-effects meta-analysis. Between-procedure comparisons were made using multilevel meta-regression. For all outcomes, more positive values indicated greater improvement; mean differences (MD) were estimated for AET and DeMeester scores, whereas standardized mean differences (SMD) were estimated for relief scores.

**Results:**

A total of 9516 patients were included with an average age of 50.8 years (SD: 6.7) with 53.7% male and an average BMI of 27.1 (SD: 2.3). The median length of follow-up across all observations was 12 months (IQR: 6–24 months; range: 0.25–120.5 months). All procedures indicated a statistically significant mean improvement in AET, relief score, and DeMeester scores (Table 1). Compared to Nissen, TIF averaged significantly less mean improvement in AET (MD: − 4.06, 95% CI: − 8.03 to − 0.09, *p* = .045) and DeMeester score (MD: − 20.60, 95% CI: − 38.33 to − 2.88, *p* = .023), whereas MSA and Toupet averaged significantly better relief scores (SMD: 0.28, 95% CI: 0.01 to 0.56, *p* = .044 and SMD = 0.17, 95% CI: 0.01 to 0.32, *p* = .034, respectively).

**Summary:**

This review advances understanding of the objective and subjective improvement of the traditional and recent surgical anti-reflux procedures used for symptomatic GERD. Overall, Nissen fundoplication demonstrated significantly better improvement with acid exposure and DeMeester score compared to MSA and TIF. However, symptom relief scores are significantly improved with Toupet fundoplication and MSA compared to other surgical treatment options.

**Supplementary Information:**

The online version contains supplementary material available at 10.1007/s00464-025-11856-5.

Gastroesophageal reflux disease (GERD) is a highly prevalent disorder [1] affecting between 10 and 30% of the western world [2–5]. Pathophysiology of reflux related to malfunction of the lower esophageal sphincter (LES) resulting in reflux of acid and gastric contents into the distal esophagus [6–10]. GERD’s substantial deleterious impact on quality of life6 is worse than those of diabetes, arthritis, and congestive heart failure [2]. GERD can lead to progression to Barrett’s and esophageal adenocarcinoma [7] coupled with chronic symptoms and complications as well as social and economic burdens pose significant challenges on the healthcare system [1, 5, 11].

Proton pump inhibitors (PPI) are considered as the first-line treatment for GERD. (R) However, when coupled with lifestyle changes, nearly 40% of patients continue to experience persistent symptoms and poor quality of life despite maximal medical therapy [2, 6, 14, 16]. With up to 90% of patients failing medical management, a surgical approach becomes more important and pertinent [16]. Laparoscopic Nissen fundoplication (LNF) is considered the most widely used surgical approach for GERD [2, 12], but it is associated with dysphagia and gas-bloating syndrome [6, 11, 13]. A number of technical modifications have been proposed to minimize the risk of these complications (anterior, Dor, and Toupet fundoplications) [11]. Laparoscopic Toupet fundoplication (LTF) has been proposed as an alternative to LNF with studies showing variable results [6, 11, 13]. LTF is reported to reduce the prevalence of post-operative dysphagia and gas-related symptoms compared to LNF [2, 6, 13]. The Transoral incisionless fundoplication (TIF) procedure has been introduced as a full-thickness gastroesophageal fundoplication via placement of polypropylene H-fasteners [1, 14]. This technique shows early signs of promise [15], although further studies for efficacy, safety, and durability remain necessary [1]. Lastly, the placement of a magnetic sphincter augmentation device (LINX) is another anti-reflux procedure that mechanically restores competency to the reflux barrier without using the gastric fundus [7]. Studies comparing surgical procedures with PPI reported significant improvement in reflux-related outcomes, but literature reporting comparisons between surgical procedures is limited.

The purpose of this meta-analysis is to compare subjective and objective outcomes among the more commonly performed anti-reflux surgical procedures (laparoscopic total Nissen fundoplication (LNF), magnetic sphincter augmentation (MSA), transoral incisionless fundoplication (TIF), Toupet fundoplication).

## Methods

This systematic review and meta-analysis were performed in accordance with the 2020 Preferred Reporting Items for Systematic reviews and Meta-Analyses statement (PRISMA 2020). The purpose of this systematic review and meta-analysis was to evaluate longitudinal follow-up of objective and subjective symptom relief following surgery for gastroesophageal reflux disease (GERD). Our study is unique in that we included all repeated follow-up measurements.

### Study eligibility

We considered only randomized controlled trials or cohort designs that included adult patients diagnosed with GERD (or GORD) who underwent a laparoscopic Nissen fundoplication (complete or total fundoplication), Toupet fundoplication (partial fundoplication, 270°), magnetic sphincter augmentation (MSA, LINX Reflux Management System), or transoral incisionless fundoplication (TIF 1.0, TIF 2.0) for GERD. Outcomes included pre-to-post-operative changes in acid exposure time (AET; percent of time with pH < 4), DeMeester score, or subjective symptom relief score as measured by the GERD-HRQL, GIQLI, QOLRAD, or VAS. As such, patients served as their own control. We excluded studies in which the patients had comorbid achalasia, reoperation for GERD, underwent a concomitant weight loss procedure, or esophagectomy.

### Search strategy

Two authors (YT, DN) systematically searched MEDLINE, Scopus, Cochrane Library, and Embase databases from inception through November 22, 2023. The search strategy was developed with the assistance of a research librarian at our institution (see Supplemental Tables S1–S4 for the complete search strategy by database). Reference lists and previous systematic reviews and meta-analyses were also searched to identify additional studies. We only considered studies in the English language. Three authors (YT, DN, RW) independently screened the titles and abstracts, with studies meeting criteria for full-text review evaluated independently by the same three authors. Any disagreement was remedied via consensus.

### Methodological quality assessments

The methodological quality was performed on all studies meeting inclusion criteria by three authors (YT, DN, RW) with disagreement remedied via consensus. Randomized controlled trials were assessed using the Jadad scale (aka, Oxford quality scoring system) [17], whereas studies using a cohort design were assessed using the Newcastle–Ottawa Scale for Cohort Studies [18]. Traditionally, a Jadad score of three or greater indicates a good-quality study; however, because blinding of the surgeon or patient was not possible in these studies, we considered a score of two or greater to indicate good quality.

### Data extraction

Three authors (YT, DN, RW) used a standardized data collection form to extract data from each study; disagreement was remedied via consensus. We extracted whether the study was a manuscript or abstract, the country in which the study was conducted, the GERD procedure, study design, total number of patients, mean baseline age, percent male, mean body mass index (BMI), and severity of esophagitis based on the Los Angeles classification system. We collected all post-procedure months to follow-up as well as the sample size at each follow-up. If a study compared the outcomes of two or more GERD procedures, outcome data were extracted separately for each procedure. For all three outcomes, we extracted the pre- and post-operative mean and an index of variability that could include standard deviation, standard error, and/or confidence interval. For studies that included median and an index of variability of either the interquartile range or range, the mean and standard deviation were estimated using methods described by Wan et al. [19].

### Statistical analyses

AET and DeMeester score are presented as unstandardized mean differences, whereas relief scores are presented as standardized mean differences using Cohen’s *d* given the differential measurement instruments used across studies. Across all outcomes, the correlation between the pre- and post-operative measurements were rarely presented. In the absence of explicit change data or the pre-to-post-operative correlation, the standard error estimate for the unstandardized or standardized mean difference was calculated assuming a correlation of 0.10, which is conservative in repeated measures data. All meta-analyses included random effects estimated using residual maximum likelihood. For each outcome, we initially estimated multilevel meta-analyses given a study could include multiple lengths of follow-up (e.g., 3 months, 1 year, 5 years) and/or multiple procedure cohorts. The decision to retain the multilevel structure was dictated by likelihood ratio test compared against a standard (inherently two-level) meta-analysis. Between-study variability estimates provided for multilevel meta-analyses are quantified as level-specific Tau-squared and Higgins–Thompson *I*^2^. We also estimated meta-regression models (multilevel versions as needed) to evaluate whether the mean differences differed between GERD procedures, study design, length of follow-up, mean baseline age, baseline percent of males, baseline mean BMI, or baseline esophagitis severity. All analyses were conducted using Stata v. 18.5 with two-tailed *p* < 0.05 used to indicate statistical significance. Note that forest plots were purposefully not created due to the number of studies and follow-up measurements.

## Results

### Study selection

**Fig. 1:**
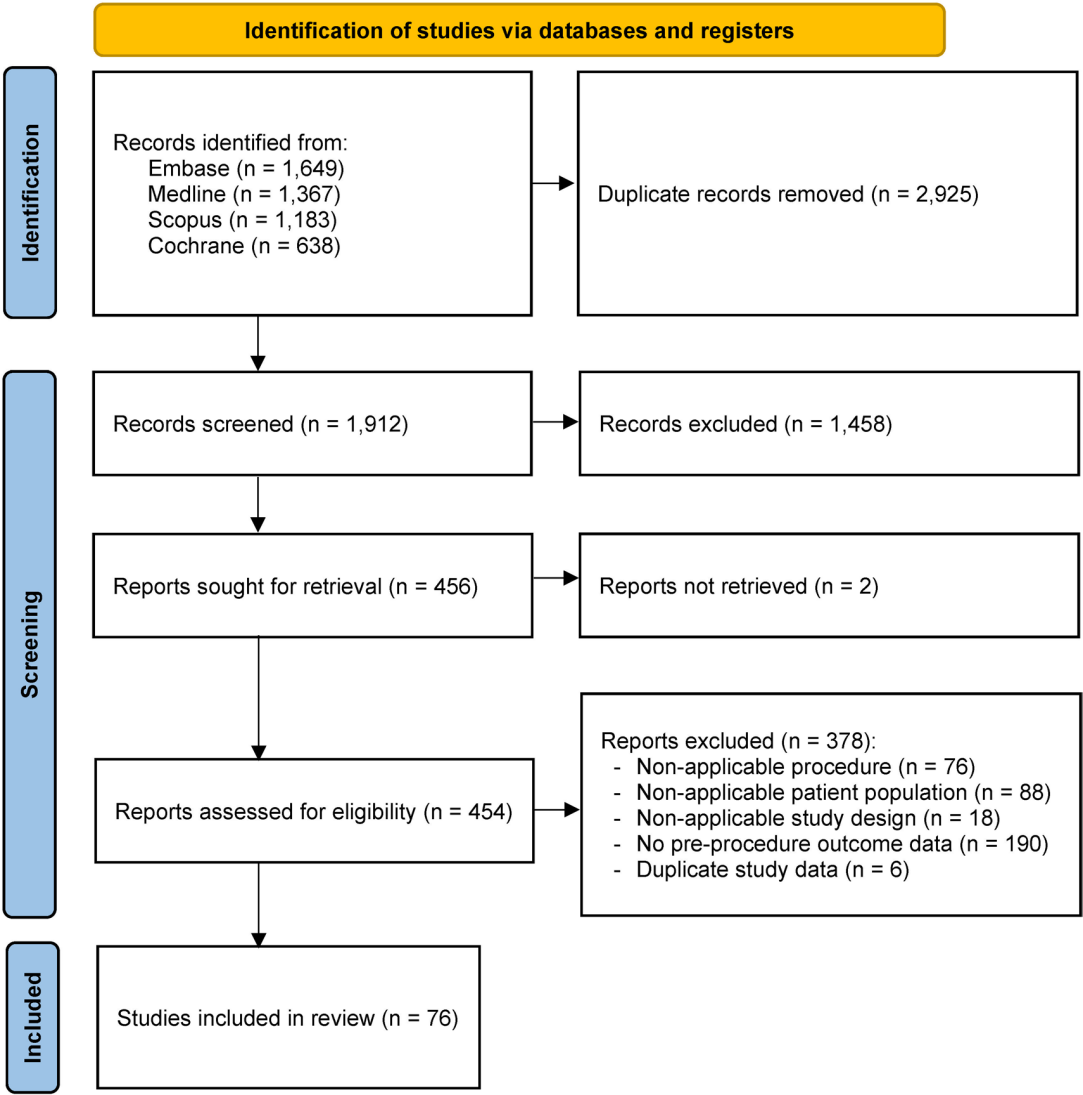
PRISMA flow chart detailing record exclusions

As shown in Fig. 1, the search yielded a total of 4837 records. After removing 2925 duplicates, titles and abstracts were reviewed which yielded 456 studies for full-text review. Primary reasons for exclusion included lack of pre-procedure outcome data and non-applicable patient population (e.g., concomitant procedure). The final systematic review and meta-analysis were based on 76 studies, of which 25 (32.9%) were randomized controlled trials and 51 (67.1%) used a cohort design. Characteristics of the studies are included in Supplemental Table S5, whereas the number of observations and lengths of follow-up by procedure are provided in Table 1.

**Table 1:** Studies, observations, and length of follow-up stratified by procedure

			Follow-up (months)	
	Studies	Observations	Median [IQR]	Range
MSA	15	27	12 [6–24]	0.5–60
Nissen	39	67	12 [12–24]	1–60
TIF	17	26	12 [3–24]	0.25–120.5
Toupet	12	21	12 [6–26]	1.5–87.6
Unspecified	6	15	12 [4–19]	1–60

### Methodological quality assessment

Of the 25 randomized controlled trials, 19 (76.0%) were indicated as good quality. Studies were scored as low quality due to lack of adequate detail about reasons for patients being lost to follow-up. Of the 51 studies using a cohort design, 35 (68.6%) were good quality. The studies rated as poor quality were primarily those that only measured the relief score outcome, which is technically self-report and therefore penalized by the Newcastle–Ottawa scale; other reasons included that the percent of patients lost to follow-up increased with longer lengths of follow-up and poor documentation of the number of patients at a given follow-up measurement. Complete methodological quality scores are provided in Supplemental Tables S6 and S7).

### Acid exposure time

A total of 26 unique studies provided 34 total follow-up observations from 2574 patients, of which 924 patients (35.9%; 11 studies) underwent a Nissen fundoplication, 915 patients (35.5%; 4 studies) underwent an MSA, 485 (18.8%; 9 studies) underwent a TIF, and 50 (1.9%; 1 study) underwent a Toupet fundoplication; the remaining 7.8% underwent an unspecified laparoscopic fundoplication. The multilevel meta-analysis model did not estimate. Significant between-study heterogeneity was observed (Tau-squared: 4.02, Higgins–Thompson *I*^2^: 93.8%). Overall, undergoing a GERD procedure provided statistically significant improvement in acid exposure time (mean improvement: 7.80, 95% CI: 6.12 to 9.48, *p* < 0.001). Although statistically significant improvement was observed for all GERD procedures, Nissen fundoplication averaged greater mean improvement compared to TIF (mean difference: 4.05, 95% CI: 0.13 to 7.96, *p* = 0.043); no other differences were statistically significant (Table 2). Further, meta-regression indicated that no study-specific variable, including longer lengths of follow-up, was associated with mean improvement (Table 3).

**Table 2:** Meta-analysis results by outcome

	Acid exposure time		Relief score		DeMeester score	
	Statistic	*p*	Statistic	*p*	Statistic	*p*
Observations						
Total	34	–	125	–	49	–
Unique studies	26	–	57	–	31	–
Procedures, n (%)						
MSA	5 (14.7)	–	25 (20.0)	–	5 (10.2)	–
Nissen	14 (41.2)	–	47 (37.6)	–	27 (55.1)	–
TIF	13 (38.2)	–	21 (16.8)	–	9 (18.4)	–
Toupet	1 (2.9)	–	18 (14.4)	–	6 (12.2)	–
Unspecified LF	1 (2.9)	–	14 (11.2)	–	2 (4.1)	–
Mean improvement, M (95% CI)						
Overall	7.80 (6.12, 9.48)	< .001	2.26 (1.96, 2.55)	< .001	30.65 (23.56, 37.73)	< .001
Procedures						
MSA	7.51 (3.86, 11.16)	< .001	2.47 (2.07, 2.86)	< .001	22.14 (5.75, 38.53)	0.008
Nissen	9.67 (7.06, 12.28)	< .001	2.18 (1.81, 2.55)	< .001	38.47 (29.69, 47.26)	< .001
TIF	5.62 (2.70, 8.54)	< .001	2.09 (1.47, 2.72)	< .001	17.75 (2.23, 33.27)	0.025
Toupet	8.38 (2.82, 13.95)	0.003	2.34 (1.97, 2.73)	< .001	33.03 (23.49, 42.57)	< .001
Procedure comparison						
Nissen vs. MSA	2.16 (− 2.33, 6.65)	0.346	− 0.29 (− 0.56, − 0.01)	0.042	16.33 (− 2.26, 34.93)	0.085
TIF vs. MSA	− 1.89 (− 6.57, 2.79)	0.428	− 0.38 (− 1.11, 0.36)	0.318	− 4.39 (− 26.96, 18.18)	0.703
Toupet vs. MSA	0.87 (− 5.79, 7.53)	0.797	− 0.12 (− 0.39, 0.15)	0.392	10.89 (− 8.08, 29.86)	0.261
Nissen vs. TIF	4.05 (0.13, 7.96)	0.043	0.09 (− 0.64, 0.82)	0.807	20.72 (2.89, 38.55)	0.023
Nissen vs. Toupet	1.28 (− 3.90, 6.47)	0.628	− 0.17 (− 0.32, − 0.01)	0.033	5.45 (0.97, 9.92)	0.017
Toupet vs. TIF	2.77 (− 3.52, 9.05)	0.389	0.26 (− 0.48, 0.99)	0.491	15.28 (− 2.94, 33.49)	0.100

For procedure comparisons, the procedures after the “vs.” is the reference group

**Table 3:** Meta-regression results by outcome

	Acid exposure time		Relief score		DeMeester score	
	Mean (95% CI)	*p*	Mean (95% CI)	*p*	Mean (95% CI)	*p*
Study design						
Cohort	7.50 (5.35, 9.64)	< .001	2.39 (2.05, 2.73)	< .001	27.95 (18.60, 37.30)	< .001
RCT	8.33 (5.55, 11.11)	< .001	1.85 (1.25, 2.44)	< .001	34.33 (23.40, 45.26)	< .001
RCT vs. Cohort	0.83 (− 2.68, 4.35)	0.642	− 0.54 (− 1.23, 0.15)	0.123	6.38 (− 8.00, 20.76)	0.385
Follow-up						
Per 1 additional month	0.03 (− 0.02, 0.09)	0.238	− 0.00 (− 0.01, − 0.00)	0.034	0.04 (− 0.05, 0.13)	0.364
Per 6 additional months	0.21 (− 0.14, 0.55)		− 0.02 (− 0.04, − 0.00)		0.24 (− 0.28, 0.76)	
Per 12 additional months	0.41 (− 0.27, 1.10)		− 0.04 (− 0.08, − 0.00)		0.48 (− 0.56, 1.52)	
By Procedure (per 12 months)						
MSA	− 0.87 (− 2.82, 1.06)	0.378	0.02 (− 0.08, 0.12)	0.732	− 0.24 (− 13.42, 12.95)	0.972
Nissen	0.36 (− 0.47, 1.19)	0.400	− 0.08 (− 0.12, − 0.02)	0.005	0.40 (− 0.71, 1.50)	0.484
TIF	1.36 (− 0.39, 3.11)	0.128	− 0.08 (− 0.27, 0.11)	0.422	0.89 (− 6.03, 7.81)	0.801
Toupet	–	–	0.08 (− 0.05, 0.21)	0.211	1.05 (− 2.69, 4.79)	0.581
Unspecified	–	–	− 0.02 (− 0.10, 0.07)	0.682	–	–
Baseline Age (per 1 additional year)	0.16 (− 0.08, 0.40)	0.182	− 0.00 (− 0.02, 0.01)	0.702	0.72 (− 0.20, 1.65)	0.125
Percent Male (per 10% more males)	0.90 (− 0.35, 2.14)	0.158	0.11 (0.02, 0.20)	0.016	− 2.21 (− 6.48, 2.05)	0.309
Baseline BMI (per 1 unit higher)	− 0.22 (− 1.07, 0.63)	0.608	− 0.06 (− 0.14, 0.02)	0.137	1.41 (− 1.62, 4.43)	0.363
25 kg/m^2^ (predicted value)	7.59 (4.66, 10.52)	–	2.21 (1.80, 2.62)	–	24.88 (13.61, 36.15)	–
30 kg/m^2^ (predicted value)	6.49 (3.09, 9.88)	–	1.90 (1.47, 2.34)	–	31.91 (18.80, 45.01)	–
35 kg/m^2^ (predicted value)	5.38 (− 1.71, 12.47)	–	1.60 (0.97, 2.33)	–	38.93 (12.97, 64.90)	–
Esophagitis (Los Angeles Classification)						
None (per 1% more patients)	− 0.04 (− 0.14, 0.07)	0.513	− 0.00 (− 0.01, 0.01)	0.605	− 0.20 (− 0.72, 0.31)	0.445
A (per 1% more patients)	0.14 (− 0.10, 0.38)	0.262	− 0.01 (− 0.04, 0.01)	0.360	− 0.51 (− 1.52, 0.50)	0.322
B (per 1% more patients)	− 0.05 (− 0.30, 0.21)	0.730	0.00 (− 0.01, 0.02)	0.715	− 0.36 (− 1.31, 0.60)	0.467
C (per 1% more patients)	0.12 (− 0.16, 0.41)	0.394	0.02 (− 0.00, 0.04)	0.064	1.74 (0.74, 2.75)	0.001
D (per 1% more patients)	0.36 (− 0.34, 1.06)	0.316	0.02 (− 0.00, 0.04)	0.061	0.78 (0.02, 1.54)	0.045

### DeMeester score

A total of 31 unique studies provided 49 total follow-up observations from 2997 patients, of which 1195 (39.9%; 17 studies) underwent Nissen fundoplication, 971 (32.4%; 4 studies) underwent an MSA, 347 (11.6%; 6 studies) underwent TIF, and 204 (6.8%; 4 studies) underwent a Toupet fundoplication; the remaining 9.3% underwent an unspecified laparoscopic fundoplication. The multilevel meta-analysis model fit significantly better than the traditional meta-analysis (− 2LL difference = 4.61, *p* = 0.032). Significant between-study heterogeneity was observed, with much of the variability observed at the study level (Tau-squared: 19.51 at level 3 and 3.00 at level 2, Higgins–Thompson *I*^2^: 95.9% at level 3 and 2.3% at level 2). Overall, undergoing a GERD procedure provided statistically significant improvement in DeMeester score (mean improvement: 30.65, 95% CI: 23.56 to 37.73, *p* < 0.001). Although statistically significant improvement was observed for all GERD procedures, Nissen fundoplication averaged greater mean improvement compared to TIF (mean difference: 20.72, 95% CI: 2.89 to 38.55, *p* = 0.023) and Toupet fundoplication (mean difference: 5.45, 95% CI: 0.97 to 9.92, *p* = 0.017); no other differences were statistically significant (Table 2). Meta-regression indicated that studies including a greater percent of patients with grade C or D esophagitis were associated with greater improvement (Table 3).

### Relief score

A total of 57 unique studies provided 125 total follow-up observations from 7146 patients, of which 2591 (36.3%; 25 studies) underwent Nissen fundoplication, 2288 (32.0%; 13 studies) underwent an MSA, 760 (10.6%; 9 studies) underwent a Toupet fundoplication, and 690 (9.7%; 13 studies) underwent TIF; the remaining 11.4% underwent an unspecified laparoscopic fundoplication. The multilevel meta-analysis model did not estimate. Significant between-study heterogeneity was observed (Tau-squared: 1.13, Higgins-Thompson *I*^2^: 97.3%). Overall, undergoing a GERD procedure provided statistically significant relief (mean improvement: 2.26, 95% CI: 1.96 to 2.55, *p* < 0.001). Although statistically significant improvement was observed for all GERD procedures, Nissen fundoplication averaged worse relief compared to MSA (mean difference: − 0.29, 95% CI: − 0.56 to − 0.01, *p* = 0.042) and Toupet fundoplication (mean difference: − 0.17, 95% CI: − 0.32 to − 0.01, *p* = 0.033); no other differences were statistically significant (Table 2). Meta-regression indicated relief was diminished as length of follow-up increased (Table 3). Specifically, for every 12-month post-procedure, relief score was lower by 0.04 standard deviations (95% CI: − 0.08 to − 0.01, *p* = 0.034), which was an effect primarily driven by Nissen fundoplication (− 0.08, 955 CI: − 0.12 to − 0.02, *p* = 0.005). Further, studies that included 10% more male patients averaged 0.11 standard deviations greater relief scores (95% CI: 0.02 to 0.20, *p* = 0.016; Table 3).

## Discussion

Anti-reflux surgery remains one of the primary treatment options for GERD and has evolved significantly over the past two decades. Fundoplication procedures such as Nissen and Toupet continue to be the mainstay of anti-reflux surgical management. However, with technological advancement, options including MSA and TIF have been increasingly performed. While the outcomes of each procedure have been individually evaluated, direct comparisons among all three options have been challenging and not well reported. This meta-analysis addressed this gap by evaluating the available literature to provide valuable insights. Our findings demonstrated that all surgical anti-reflux procedures (LNF, Toupet, TIF, and MSA) significantly improved DeMeester scores, AET, and subjective symptom relief scores. The subjective and objective improvements are durable and persisted over long-term follow-up. Our results were similar to previously published long-term outcomes after anti-reflux surgery [20]. Although there have been questions raised regarding the long-term durability of anti-reflux surgery, our results reinforce the role of anti-reflux surgery with both objective and subjective improvements.

Overall, LNF consistently demonstrated a significant reduction in DeMeester scores and post-operative acid exposure times in both short- and long-term follow-up. These findings were similar to the previously published long-term studies, reaffirming the durability of LNF in reflux control. LNF also demonstrated a superior reduction in AET when compared to TIF and achieved greater improvements in DeMeester scores when compared to both TIF and Toupet procedures. The acid measurement metrics reflected similar improvements as it restores the lower esophageal sphincter competency compared to the previously published literature [21, 22]. Compared to TIF and Toupet fundoplication, LNF achieves superior improvements in objective reflux control by effectively restoring lower esophageal sphincter competency, reinforcing its role as a highly effective surgical option for GERD management. These results may be contradictory to the recent meta-analysis performed by Li et al. [23] and randomized controlled trials by Koch et al. [24], which compared LNF and Toupet and demonstrated either no difference in short term or small but insignificant difference in reflux studies. Compared to the previous study, our results included more LNF patients while the numbers for Toupet were fewer than the previous study; however, our study additionally incorporates all available follow-up measurements from patients postoperatively. Our results showed that LNF was associated with better acid control compared to the partial fundoplication; either Toupet fundoplication or TIF. Our results comparing LNF with LTF should be interpreted with caution as our methodological selection has led to unbalanced groups. Although our results showed better acid control, the determination of surgical approach and type of fundoplication continue to be dependent on multiple factors, including severity of reflux and presence of additional symptoms like dysphagia, which might ultimately have an impact on relief scores and should be addressed in future studies.

Esophagitis is usually indicative of uncontrolled acid reflux and may lead to complications like stricture. Esophagitis was not a primary outcome of this study; however, stratification by patient characteristics, including esophagitis, presented an interesting trend. Surgical anti-reflux interventions have been reported to result in proportionally greater improvement depending on the severity of esophagitis. Our results also showed that patients with more severe esophagitis had the greatest improvements in DeMeester scores. Tsuboi et al. [25] reported a similar finding, demonstrating a significant increase in the therapeutic effect of LNF in patients with worse preoperative reflux control, as measured by multichannel intraluminal impedance pH (MII-pH) and high-resolution manometry (HRM). Consistent with previous observations on AET, the greater reduction in DeMeester score following LNF supports its use in patients requiring more aggressive intervention, particularly those with moderate-to-severe esophagitis. Overall, the improved efficacy of LNF in AET reduction underscores its role in patients who need robust acid suppression, especially in patients with esophagitis.

Anti-reflux surgery outcomes are often measured using long-term symptom relief scores. Tracking patient-reported outcomes as the metric for long-term symptom relief remains the primary method of understanding and improving patient satisfaction. Symptomatic relief in GERD is utilized as a metric for objective reflux control measures. However, subjective symptom relief scores indicated that MSA and Toupet fundoplication provided superior symptom relief compared to LNF. Symptom relief scores like GERD-HRQL questionnaires often depend on patient’s perceived relief from symptoms. While LNF is highly effective in acid suppression, Aiolfi et al. [26] noted its association with higher rates of post-operative dysphagia and bloating, contributing to lower perceived symptom relief. Notably, our results revealed a decline in symptom improvement scores over time, primarily driven by LNF patients. This deterioration in long-term relief aligns with Aiolfi et al. [26] and Guidozzi et al. [27], linking LNF to increased rates of dysphagia and bloating, potentially explaining the discrepancy between its greater objective reflux control yet lower patient relief. AET and DeMeester scores serve as objective indicators of acid suppression, whereas patient-reported relief scores reflect perceived symptom control, offering a subjective measure of both short- and long-term procedural success. The discrepancy between these objective and subjective outcomes underscores the need to balance procedural efficacy and acid suppression with patient satisfaction when making surgical decisions. Overall patient-reported outcomes are cornerstone for long-term success of procedure. These should be taken into consideration before performing anti-reflux surgery and matching patient expectations with objective outcomes will improve overall outcomes.

Furthermore, meta-regressions evaluating patient characteristics demonstrated that studies with a higher proportion of male patients reported better symptom relief. Although the underlying reason for this trend remains unclear, prior research has identified male sex as a strong predictor of greater post-operative satisfaction [28–30]. Patient relief scores are significantly better in males irrespective of acid exposure times and DeMeester scores which remained similar across the study population. Our results also indicated that BMI categories were not associated with any significant difference in acid exposure time, DeMeester score, or patient relief scores. There has been significant controversy regarding the impact of BMI on acid reflux severity and potential impact on outcomes of anti-reflux surgery. The results of this study suggest no significant differences in acid exposure measurements when stratified with BMI. However, our results do not imply that anti-reflux procedures in higher BMI patients produce similar results compared to lower BMI patients.

A key strength of this study is its comprehensive approach, incorporating multiple follow-up time points into the meta-analysis. To our knowledge, this is the first meta-analysis to provide such a longitudinal perspective, offering a more nuanced comparison of these commonly performed procedures. These findings highlight the importance of considering both objective reflux control and patient-reported outcomes when selecting the most appropriate surgical intervention for GERD. Further research with extended follow-up is warranted to better understand the longer-term trade-offs between reflux control and post-operative quality of life.

This study also bears several limitations which include lack of hernia characteristics, technical details of hernia repair, and variations in TIF and MSA procedures within the meta-regression. Our analysis also did not analyze post-operative complications such as dysphagia and bloating which could impact post-operative quality of life questionnaire scores. Outcome differences between TIF1.0 and TIF2.0 or cTIF were not separated in this study.

In conclusion, this meta-analysis has increased our understanding of pre- and post-operative subjective and objective improvement from various surgical interventions. Overall, Nissen fundoplication demonstrated significantly better improvement with acid exposure and DeMeester score compared to MSA and TIF. However, symptom relief scores are significantly improved with Toupet fundoplication and MSA compared to other surgical treatment options.

## Supplementary Information

Below is the link to the electronic supplementary material.

Supplementary file1 (DOCX 541 KB)

## Abbreviations

GERD: Gastroesophageal reflux disease
MSA: Magnetic sphincter augmentation
AET: Acid exposure time
TIF: Transoral incisionless fundoplication
LNF: Laparoscopic Nissen fundoplication
TF: Toupet fundoplication
LES: Lower esophageal sphincter
QoL: Quality of life
