# Supplementary material for: Fundoplication significantly improves objective and subjective reflux outcomes—a meta-analysis
Source: Surg Endosc. 2025 May 29;39(7):4496–504. doi: 10.1007/s00464-025-11856-5 (PMC12222424; doi:10.1007/s00464-025-11856-5)
Supplement: Supplementary file 1 — Supplementary file1 (DOCX 541 KB) [file 464_2025_11856_MOESM1_ESM.docx]

**Online Supplement**

**Table S1.** MEDLINE search strategy 2

**Table S2.** Embase search strategy 5

**Table S3.** Cochrane library search strategy 7

**Table S4.** Scopus search strategy 8

**Table S5.** Study Characteristics 9

**Table S6.** Jadad scores for randomized controlled trials 12

**Table S7.** Newcastle-Ottawa scores for cohort designs 13

**Table S1.** MEDLINE search strategy

**
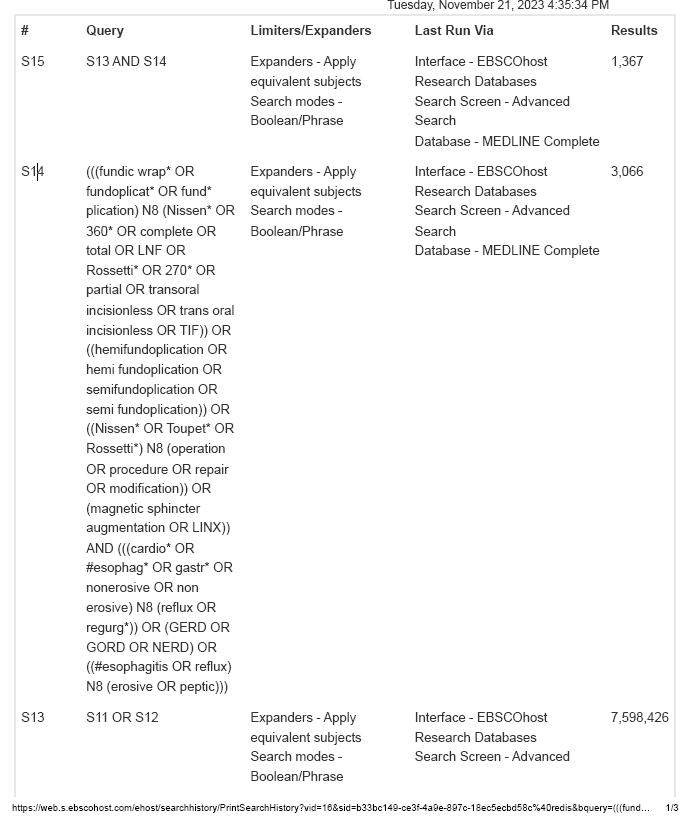
**

**
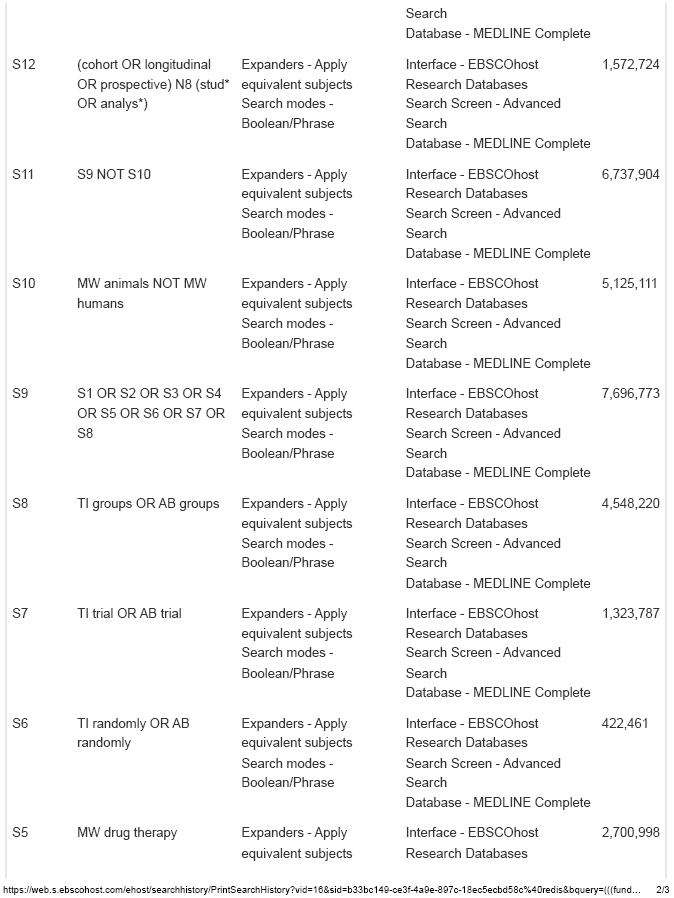
**

**
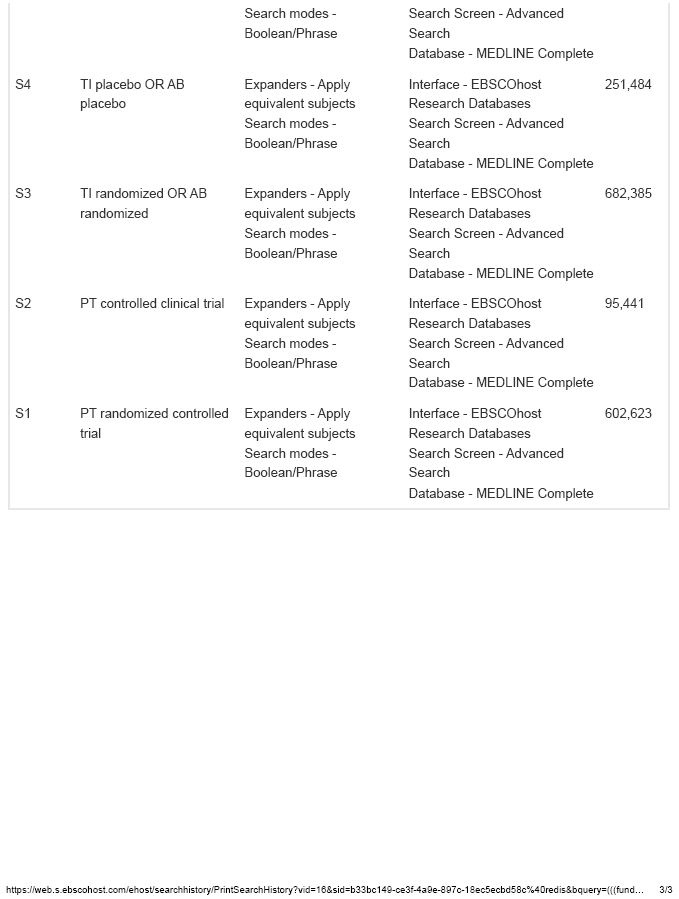
**

**Table S2**. Embase search strategy


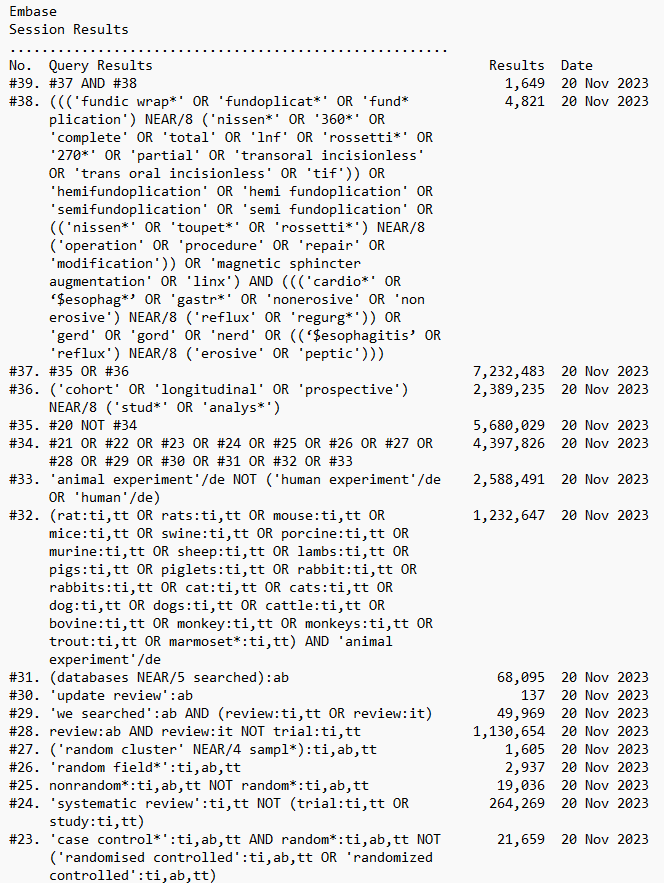


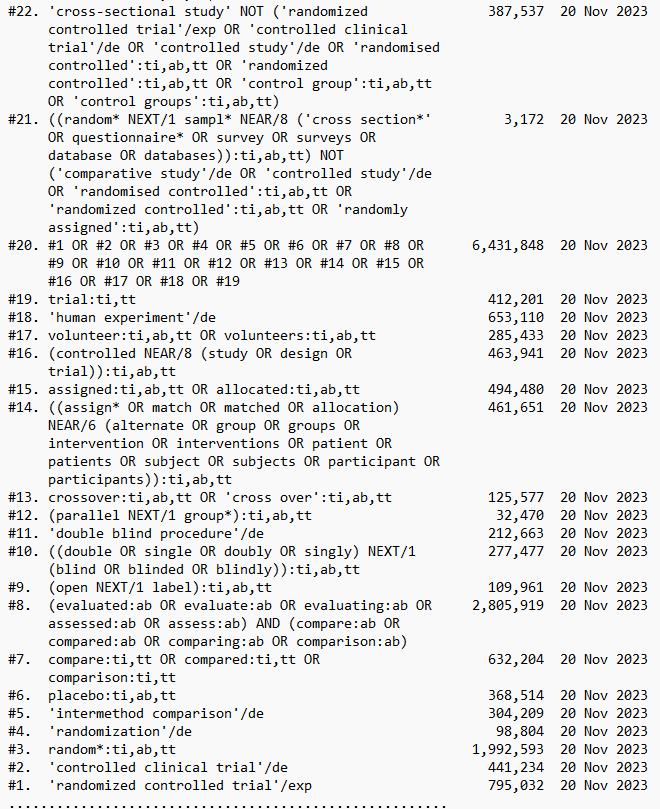


**Table S3.** Cochrane library search strategy


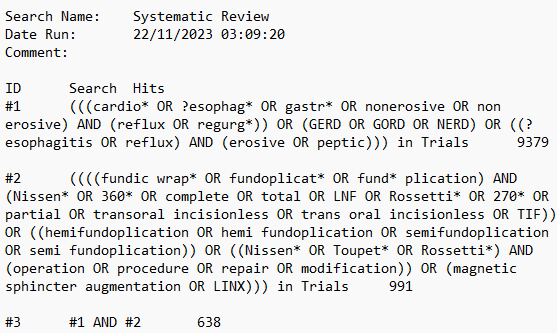


**Table S4.** Scopus search strategy

**
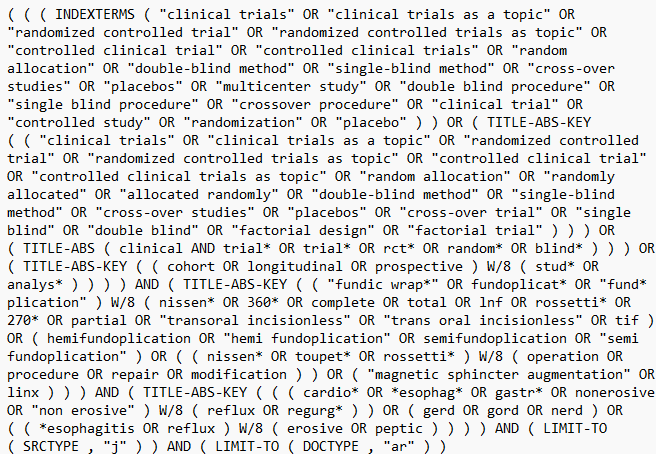
**

**Table S5.** Study Characteristics

| **Author** | **Year** | **Country** | **Procedure** | **Design** | **N_Total** | **FollowUp** | **Age_Base** | **Male_Base** | **BMI_Base** | **EsoNone_Base** | **EsoA_Base** | **EsoB_Base** | **EsoC_Base** | **EsoD_Base** | **AET** | **Relief** | **DeMeester** |
| --- | --- | --- | --- | --- | --- | --- | --- | --- | --- | --- | --- | --- | --- | --- | --- | --- | --- |
| Allen et al. | 1998 | Canada | Nissen | Cohort | 381 | 6 | 46 | 36.2 |  |  |  |  |  |  | 1 | 1 | 0 |
| Allen et al. | 1998 | Canada | Nissen | Cohort | 381 | 24 | 46 | 36.2 |  |  |  |  |  |  | 1 | 1 | 0 |
| Heikkinen et al. | 2000 | Finland | Nissen | RCT | 21 | 24.0 | 48.0 | 66.0 | 27.2 | 2.0 | 18.0 | 26.0 | 42.0 | 12.0 | 0 | 1 | 0 |
| Kamolz et al. | 2000 | Austria | Nissen | Cohort | 107 | 1.5 | 52.1 | 55.4 |  | 15.0 | 7.0 | 9.0 | 17.0 | 52.0 | 0 | 1 | 0 |
| Kamolz et al. | 2000 | Austria | Toupet | Cohort | 68 | 1.5 | 52.1 | 55.4 |  | 15.0 | 7.0 | 9.0 | 17.0 | 52.0 | 0 | 1 | 0 |
| Kamolz et al. | 2000 | Austria | Nissen | Cohort | 107 | 3.0 | 52.1 | 55.4 |  | 15.0 | 7.0 | 9.0 | 17.0 | 52.0 | 0 | 1 | 1 |
| Kamolz et al. | 2000 | Austria | Toupet | Cohort | 68 | 3.0 | 52.1 | 55.4 |  | 15.0 | 7.0 | 9.0 | 17.0 | 52.0 | 0 | 1 | 1 |
| Kamolz et al. | 2000 | Austria | Nissen | Cohort | 107 | 12.0 | 52.1 | 55.4 |  | 15.0 | 7.0 | 9.0 | 17.0 | 52.0 | 0 | 1 | 1 |
| Kamolz et al. | 2000 | Austria | Toupet | Cohort | 68 | 12.0 | 52.1 | 55.4 |  | 15.0 | 7.0 | 9.0 | 17.0 | 52.0 | 0 | 1 | 1 |
| Slim et al. | 2000 | France | Toupet | Cohort | 50 | 3.0 | 49.9 | 60.0 |  |  |  |  |  |  | 0 | 1 | 0 |
| Slim et al. | 2000 | France | Toupet | Cohort | 50 | 12.0 | 49.9 | 60.0 |  |  |  |  |  |  | 0 | 1 | 0 |
| Fibbe et al. | 2001 | Germany | LF | RCT | 200 | 4 | 56 | 60.5 |  |  |  |  |  |  | 1 | 0 | 1 |
| Kamolz et al. | 2001 | Austria | Nissen | Cohort | 191 | 3.0 | 51.2 | 62.3 |  | 2.1 | 5.2 | 20.4 | 30.8 | 41.3 | 0 | 1 | 1 |
| Kamolz et al. | 2001 | Austria | Nissen | Cohort | 191 | 12.0 | 51.2 | 62.3 |  | 2.1 | 5.2 | 20.4 | 30.8 | 41.3 | 0 | 1 | 1 |
| Lochegnies et al. | 2001 | Belgium | Nissen | Cohort | 31 | 36.0 | 39.0 | 74.1 |  |  |  |  |  |  | 0 | 1 | 0 |
| Granderath et al. | 2002 | Austria | Nissen | Cohort | 150 | 3.0 | 51.8 | 68.0 |  |  |  |  |  |  | 0 | 1 | 1 |
| Granderath et al. | 2002 | Austria | Nissen | Cohort | 150 | 12.0 | 51.8 | 68.0 |  |  |  |  |  |  | 0 | 1 | 1 |
| Granderath et al. | 2002 | Austria | Nissen | Cohort | 150 | 36.0 | 51.8 | 68.0 |  |  |  |  |  |  | 0 | 1 | 1 |
| Zugel et al. | 2002 | Germany | Nissen | Cohort | 40 | 19.0 | 45.2 | 65.0 |  | 15.0 | 30.0 | 25.0 | 20.0 | 10.0 | 0 | 1 | 0 |
| Zugel et al. | 2002 | Germany | Toupet | Cohort | 122 | 19.0 | 46.6 | 66.0 |  | 20.5 | 39.3 | 22.1 | 13.1 | 4.9 | 0 | 1 | 0 |
| Kamolz et al. | 2003 | Austria | Nissen | Cohort | 249 | 3.0 | 50.5 | 55.4 |  |  | 28.2 | 39.7 | 32.2 |  | 0 | 1 | 0 |
| Kamolz et al. | 2003 | Austria | Nissen | Cohort | 249 | 12.0 | 50.5 | 55.4 |  |  | 28.2 | 39.7 | 32.2 |  | 0 | 1 | 0 |
| Kamolz et al. | 2003 | Austria | Nissen | Cohort | 249 | 36.0 | 50.5 | 55.4 |  |  | 28.2 | 39.7 | 32.2 |  | 0 | 1 | 0 |
| Takeyama et al. | 2004 | Japan | Nissen | Cohort | 23 | 23 | 66.3 | 30.4 |  | 0 | 17.39 | 17.39 | 47.826 | 17.39 | 1 | 0 | 0 |
| Granderath et al. | 2005 | Austria | Nissen | RCT | 100 | 3.0 | 48.5 | 62.0 | 28.0 |  |  |  |  |  | 0 | 0 | 1 |
| Granderath et al. | 2005 | Austria | Nissen | RCT | 100 | 12.0 | 48.5 | 62.0 | 28.0 |  |  |  |  |  | 0 | 0 | 1 |
| Mahon et al. | 2005 | United Kingdom | Nissen | RCT | 104 | 3 | 48 | 65 |  |  |  |  |  |  | 1 | 1 | 1 |
| Mahon et al. | 2005 | United Kingdom | Nissen | RCT | 104 | 12 | 48 | 65 |  |  |  |  |  |  | 0 | 1 | 0 |
| Neumayer et al. | 2005 | Austria | Nissen | Cohort | 213 | 3.0 | 49.0 | 60.1 | 27.6 | 4.7 | 10.8 | 16.9 | 7.5 | 26.3 | 0 | 1 | 0 |
| Neumayer et al. | 2005 | Austria | Nissen | Cohort | 213 | 12.0 | 49.0 | 60.1 | 27.6 | 4.7 | 10.8 | 16.9 | 7.5 | 26.3 | 0 | 1 | 0 |
| Pidoto et al. | 2006 | Italy | Nissen | Cohort | 25 | 6.0 | 43.8 | 70.0 |  | 0.0 | 52.0 | 40.0 | 4.0 | 4.0 | 1 | 1 | 1 |
| Pidoto et al. | 2006 | Italy | Nissen | Cohort | 25 | 120.5 | 54.3 | 70.0 |  | 0.0 | 52.0 | 40.0 | 4.0 | 4.0 | 1 | 1 | 1 |
| Rothstein et al. | 2006 | USA | EFTP | RCT | 78 | 3.0 | 48.1 | 45.0 |  |  |  |  |  |  | 0 | 1 | 0 |
| Zehetner et al. | 2006 | Switzerland | Nissen | Cohort | 100 | 2.0 | 50.4 | 63.0 |  |  |  |  |  |  | 1 | 0 | 0 |
| Balci et al. | 2007 | Turkey | Nissen | Cohort | 60 | 1.0 | 39.0 | 33.0 |  |  |  |  |  |  | 0 | 1 | 0 |
| Balci et al. | 2007 | Turkey | Nissen | Cohort | 60 | 6.0 | 39.0 | 33.0 |  |  |  |  |  |  | 0 | 1 | 0 |
| Kosek et al. | 2009 | Austria | Nissen | RCT | 41 | 60.0 | 50.0 | 73.2 |  |  |  |  |  |  | 0 | 0 | 1 |
| Demyttenaere et al. | 2010 | USA | TIF | Cohort | 26 | 10.0 | 45.0 | 38.4 | 28.0 |  |  |  |  |  | 0 | 1 | 0 |
| Shaw et al. | 2010 | South Africa | Nissen | RCT | 50 | 59.76 | 45.2 | 62 | 29.3 |  |  |  |  |  | 1 | 0 | 1 |
| Shaw et al. | 2010 | South Africa | Toupet | RCT | 50 | 55.18 | 45.6 | 58 | 29.2 |  |  |  |  |  | 1 | 0 | 1 |
| Testoni et al. | 2010 | Italy | TIF | Cohort | 20 | 6.0 | 45.0 | 60.0 | 24.0 |  |  |  |  |  | 0 | 1 | 1 |
| Anvari et al. | 2011 | Canada | Nissen | RCT | 51 | 36 | 42.9 | 55.77 |  |  |  |  |  |  | 1 | 1 | 0 |
| Brillantino et al. | 2011 | Italy | Nissen | Cohort | 35 | 12.0 | 44.6 | 45.7 |  | 28.5 |  |  |  |  | 1 | 0 | 0 |
| Koch et al. | 2011 | Austria | Nissen | RCT | 50 | 3 | 49.7 | 60 | 28.59 |  |  |  |  |  | 0 | 1 | 1 |
| Koch et al. | 2011 | Austria | Toupet | RCT | 50 | 3 | 52.36 | 68 | 27.6 |  |  |  |  |  | 0 | 1 | 1 |
| Aprea et al. | 2012 | Italy | Nissen | Cohort | 36 | 1 | 70.83 | 58.33 |  |  |  |  |  |  | 1 | 0 | 0 |
| Aprea et al. | 2012 | Italy | Nissen | Cohort | 36 | 6 | 70.83 | 58.33 |  |  |  |  |  |  | 1 | 0 | 0 |
| Bell et al. | 2012 | USA | TIF 2.0 | Cohort | 100 | 6 | 53 | 35 | 26.4 | 48 | 14 | 34 | 4 | 0 | 1 | 1 | 1 |
| Cao et al. | 2012 | China | Nissen | RCT | 50 | 6 | 59.1 | 42 | 29.7 | 14 | 26 | 8 | 30 | 22 | 0 | 0 | 1 |
| Cao et al. | 2012 | China | Partial | RCT | 50 | 6 | 57.2 | 32 | 28.6 | 12 | 22 | 18 | 32 | 16 | 0 | 0 | 1 |
| Cao et al. | 2012 | China | Nissen | RCT | 50 | 12 | 59.1 | 42 | 29.7 | 14 | 26 | 8 | 30 | 22 | 0 | 0 | 1 |
| Cao et al. | 2012 | China | Partial | RCT | 50 | 12 | 57.2 | 32 | 28.6 | 12 | 22 | 18 | 32 | 16 | 0 | 0 | 1 |
| Cao et al. | 2012 | China | Nissen | RCT | 50 | 24 | 59.1 | 42 | 29.7 | 14 | 26 | 8 | 30 | 22 | 0 | 0 | 1 |
| Cao et al. | 2012 | China | Partial | RCT | 50 | 24 | 57.2 | 32 | 28.6 | 12 | 22 | 18 | 32 | 16 | 0 | 0 | 1 |
| Cao et al. | 2012 | China | Nissen | RCT | 50 | 60 | 59.1 | 42 | 29.7 | 14 | 26 | 8 | 30 | 22 | 0 | 0 | 1 |
| Cao et al. | 2012 | China | Partial | RCT | 50 | 60 | 57.2 | 32 | 28.6 | 12 | 22 | 18 | 32 | 16 | 0 | 0 | 1 |
| Lipham et al. | 2012 | USA | MSA | Cohort | 44 | 12 | 42.8 | 59.1 |  |  |  |  |  |  | 0 | 1 | 0 |
| Lipham et al. | 2012 | USA | MSA | Cohort | 44 | 24 | 42.8 | 59.1 |  |  |  |  |  |  | 0 | 1 | 0 |
| Lipham et al. | 2012 | USA | MSA | Cohort | 44 | 36 | 42.8 | 59.1 |  |  |  |  |  |  | 0 | 1 | 0 |
| Lipham et al. | 2012 | USA | MSA | Cohort | 44 | 48 | 42.8 | 59.1 |  |  |  |  |  |  | 1 | 1 | 0 |
| Muls et al. | 2012 | Belgium | TIF | Cohort | 54 | 12 | 43 | 65 | 25.1 |  |  |  |  |  | 1 | 1 | 0 |
| Muls et al. | 2012 | Belgium | TIF | Cohort | 54 | 36 | 43 | 65 | 25.1 |  |  |  |  |  | 1 | 1 | 0 |
| Koch et al. | 2013 | Austria | Nissen | RCT | 62 | 13.03 | 50.32 | 56.45 | 28.18 |  |  |  |  |  | 0 | 1 | 1 |
| Koch et al. | 2013 | Austria | Toupet | RCT | 63 | 13.03 | 51.87 | 68.25 | 27.32 |  |  |  |  |  | 0 | 1 | 1 |
| Bell et al. | 2014 | USA | TIF 2.0 | Cohort | 127 | 6 | 53.1 | 32.3 | 26.8 | 45.7 | 14.17 | 35.43 | 4.72 | 0 | 1 | 0 | 1 |
| Bell et al. | 2014 | USA | TIF 2.0 | Cohort | 127 | 12 | 53.1 | 32.3 | 26.8 | 45.7 | 14.17 | 35.43 | 4.72 | 0 | 1 | 0 | 1 |
|  |  |  |  |  |  |  |  |  |  |  |  |  |  |  |  |  |  |
|  |  |  |  |  |  |  |  |  |  |  |  |  |  |  |  |  |  |
| **Author** | **Year** | **Country** | **Procedure** | **Design** | **N_Total** | **FollowUp** | **Age_Base** | **Male_Base** | **BMI_Base** | **EsoNone_Base** | **EsoA_Base** | **EsoB_Base** | **EsoC_Base** | **EsoD_Base** | **AET** | **Relief** | **DeMeester** |
| Bell et al. | 2014 | USA | TIF 2.0 | Cohort | 127 | 24 | 53.1 | 32.3 | 26.8 | 45.7 | 14.17 | 35.43 | 4.72 | 0 | 1 | 1 | 1 |
| Ozmen et al. | 2014 | Turkey | Nissen | Cohort | 60 | 12.0 | 42.0 | 56.7 | 25.9 |  |  |  |  |  | 0 | 1 | 0 |
| Wilson et al. | 2014 | USA | TIF 2.0 | Cohort | 100 | 12 | 53 | 35 | 26.4 | 48 | 14 | 34 | 4 | 0 | 0 | 1 | 0 |
| Luketina et al. | 2015 | Austria | LF | Cohort | 80 | 14.7 | 50.7 | 56.2 | 27.9 |  |  |  |  |  | 0 | 1 | 1 |
| Muller-Stich et al. | 2015 | Germany/Switzerland | Nissen | RCT | 44 | 3 | 49.5 | 50 | 28 | 22.7 | 36.4 | 27.3 | 9.1 | 4.5 | 0 | 1 | 1 |
| Muller-Stich et al. | 2015 | Germany/Switzerland | Nissen | RCT | 44 | 12 | 49.5 | 50 | 28 | 22.7 | 36.4 | 27.3 | 9.1 | 4.5 | 0 | 1 | 0 |
| Muller-Stich et al. | 2015 | Germany/Switzerland | Nissen | RCT | 44 | 36 | 49.5 | 50 | 28 | 22.7 | 36.4 | 27.3 | 9.1 | 4.5 | 0 | 1 | 0 |
| Rinsma et al. | 2015 | Belgium | TIF | RCT | 32 | 6.0 |  |  |  |  |  |  |  |  | 1 | 1 | 0 |
| Roy-Shapira et al. | 2015 | India | TIF | Cohort | 13 | 1.5 | 46.0 | 84.6 |  |  |  |  |  |  | 1 | 0 | 0 |
| Saino et al. | 2015 | International | MSA | Cohort | 44 | 60.0 | 42.8 |  | 25.7 |  |  |  |  |  | 0 | 1 | 0 |
| Testoni et al. | 2015 | Italy | TIF 2.0 | Cohort | 50 | 6.0 | 45.0 | 70.0 | 22.0 |  | 20.0 | 2.0 |  |  | 0 | 0 | 1 |
| Testoni et al. | 2015 | Italy | TIF 2.0 | Cohort | 50 | 24.0 | 45.0 | 70.0 | 22.0 |  | 20.0 | 2.0 |  |  | 0 | 1 | 1 |
| Testoni et al. | 2015 | Italy | TIF 2.0 | Cohort | 50 | 36.0 | 45.0 | 70.0 | 22.0 |  | 20.0 | 2.0 |  |  | 0 | 1 | 0 |
| Wang et al. | 2015 | China | Nissen | RCT | 43 | 36 | 57 | 46.5 | 23.5 |  |  |  |  |  | 0 | 0 | 1 |
| Wang et al. | 2015 | China | Toupet | RCT | 41 | 36 | 57 | 58.5 | 23.5 |  |  |  |  |  | 0 | 0 | 1 |
| Witteman et al. | 2015 | Netherlands | TIF 2.0 | RCT | 60 | 6 | 42.4 | 60 | 26 | 68 | 17 | 15 | 0 | 0 | 1 | 1 | 0 |
| Witteman et al. | 2015 | Netherlands | TIF 2.0 | RCT | 60 | 12 | 42.4 | 60 | 26 | 68 | 17 | 15 | 0 | 0 | 1 | 1 | 0 |
| Ospanov et al. | 2016 | Kazakhstan | Nissen | RCT | 58 | 12.0 | 46.3 | 41.3 | 35.2 | 0.0 | 34.4 | 37.9 | 19.0 | 8.6 | 1 | 1 | 1 |
| Chiu et al. | 2017 | China | Nissen | RCT | 36 |  | 52.0 | 61.1 |  |  |  |  |  |  | 0 | 0 | 1 |
| Schneider et al. | 2017 | USA | Nissen | RCT | 46 | 12.9 | 46.0 | 36.9 |  | 28.4 |  |  |  |  | 0 | 1 | 0 |
| Trad et al. | 2017 | USA | TIF 2.0 | RCT | 60 | 34.0 | 51.5 | 45.0 | 28.5 | 0.0 | 5.0 | 95.0 | 0.0 | 0.0 | 0 | 1 | 0 |
| Castelijns et al. | 2018b | Netherlands | TIF | RCT | 45 | 87.6 |  |  |  |  |  |  |  |  | 0 | 1 | 0 |
| Ilyashenko et al. | 2018 | Ukraine | Nissen | Cohort | 98 | 48.0 | 63.0 | 32.7 |  |  |  |  |  |  | 0 | 1 | 1 |
| Kalapala et al. | 2018 | India | EFTP | RCT | 24 | 3.0 | 37.0 |  |  |  |  |  |  |  | 0 | 1 | 0 |
| Richards et al. | 2018 | USA | LF | Cohort | 6 | 12.0 | 61.7 |  |  |  |  |  |  |  | 0 | 1 | 0 |
| Richards et al. | 2018 | USA | MSA | Cohort | 32 | 12.0 | 54.5 |  |  |  |  |  |  |  | 0 | 1 | 0 |
| Ihde et al. | 2019 | USA | TIF 2.0 | Cohort | 29 | 9.9 |  |  |  |  |  |  |  |  | 0 | 1 | 0 |
| Janu et al. | 2019 | USA | TIF 2.0 | Cohort | 99 | 6.0 | 53.2 | 45.0 | 30.0 |  |  |  |  |  | 0 | 1 | 0 |
| Janu et al. | 2019 | USA | TIF 2.0 | Cohort | 99 | 12.0 | 53.2 | 45.0 | 30.0 |  |  |  |  |  | 0 | 1 | 0 |
| Kothari et al. | 2019 | USA | MSA | Cohort | 100 | 12.0 |  |  |  |  |  |  |  |  | 0 | 1 | 1 |
| Li et al. | 2019 | China | Nissen | RCT | 61 | 6.0 | 53.0 | 59.0 | 23.8 | 63.9 | 18.0 | 9.8 | 6.6 | 1.6 | 1 | 0 | 1 |
| Louie et al. | 2019 | USA | MSA | Cohort | 200 | 12.0 | 48.5 | 51.0 | 27.4 | 74.7 | 18.2 | 5.6 | 1.0 | 0.5 | 1 | 1 | 1 |
| Park et al. | 2019 | Korea | Nissen | Cohort | 51 | 0.3 | 53.3 | 60.8 | 23.4 | 43.8 | 43.8 | 10.4 | 2.1 | 0.0 | 0 | 1 | 0 |
| Park et al. | 2019 | Korea | Nissen | Cohort | 51 | 3.0 | 53.3 | 60.8 | 23.4 | 43.8 | 43.8 | 10.4 | 2.1 | 0.0 | 0 | 1 | 0 |
| Tezcaner et al. | 2019 | Turkey | Nissen | Cohort | 32 | 28.0 | 46.7 | 53.0 |  | 0.0 | 18.8 | 50.0 | 25.0 | 6.3 | 0 | 1 | 1 |
| Ayazi et al. | 2020a | USA | MSA | Cohort | 380 | 11.5 | 55.2 | 36.1 | 29.1 |  |  |  |  |  | 0 | 1 | 0 |
| Ayazi et al. | 2020b | USA | MSA | Cohort | 553 | 10.3 | 54.7 | 38.2 | 29.0 |  |  |  |  |  | 0 | 1 | 1 |
| Ferrari et al. | 2020 | Italy | MSA | Cohort | 335 | 12.0 | 45.0 | 66.1 | 26.0 | 80.6 | 9.9 | 8.1 | 0.9 | 0.6 | 1 | 0 | 1 |
| Turner et al. | 2020 | USA | LF | Cohort | 78 | 0.5 | 51.3 | 66.6 | 29.8 |  |  |  |  |  | 0 | 1 | 0 |
| Turner et al. | 2020 | USA | LF | Cohort | 78 | 2.0 | 51.3 | 66.6 | 29.8 |  |  |  |  |  | 0 | 1 | 0 |
| Turner et al. | 2020 | USA | LF | Cohort | 78 | 6.0 | 51.3 | 66.6 | 29.8 |  |  |  |  |  | 0 | 1 | 0 |
| Turner et al. | 2020 | USA | LF | Cohort | 78 | 12.0 | 51.3 | 66.6 | 29.8 |  |  |  |  |  | 0 | 1 | 0 |
| Turner et al. | 2020 | USA | LF | Cohort | 78 | 24.0 | 51.3 | 66.6 | 29.8 |  |  |  |  |  | 0 | 1 | 0 |
| Bonavina et al. | 2021 | Europe | LF | Cohort | 166 | 12.0 | 56.3 | 49.4 | 27.8 | 40.9 | 29.6 | 16.4 | 8.2 | 5.0 | 0 | 1 | 0 |
| Bonavina et al. | 2021 | Europe | MSA | Cohort | 465 | 12.0 | 46.6 | 63.7 | 25.7 | 53.0 | 31.7 | 13.5 | 1.1 | 0.7 | 0 | 1 | 0 |
| Bonavina et al. | 2021 | Europe | LF | Cohort | 166 | 24.0 | 56.3 | 49.4 | 27.8 | 40.9 | 29.6 | 16.4 | 8.2 | 5.0 | 0 | 1 | 0 |
| Bonavina et al. | 2021 | Europe | MSA | Cohort | 465 | 24.0 | 46.6 | 63.7 | 25.7 | 53.0 | 31.7 | 13.5 | 1.1 | 0.7 | 0 | 1 | 0 |
| Bonavina et al. | 2021 | Europe | LF | Cohort | 166 | 36.0 | 56.3 | 49.4 | 27.8 | 40.9 | 29.6 | 16.4 | 8.2 | 5.0 | 0 | 1 | 0 |
| Bonavina et al. | 2021 | Europe | MSA | Cohort | 465 | 36.0 | 46.6 | 63.7 | 25.7 | 53.0 | 31.7 | 13.5 | 1.1 | 0.7 | 0 | 1 | 0 |
| Ferrari et al. | 2021 | Italy | MSA | Cohort | 336 | 50.4 | 45.5 | 67.2 | 24.9 |  |  |  |  |  | 1 | 1 | 1 |
| He et al. | 2021 | China | Nissen | Cohort | 51 | 1.0 | 53.2 | 70.6 | 22.9 |  |  |  |  |  | 0 | 1 | 0 |
| He et al. | 2021 | China | Nissen | Cohort | 51 | 2.0 | 53.2 | 70.6 | 22.9 |  |  |  |  |  | 0 | 1 | 0 |
| Roccato et al. | 2021 | USA | TIF | Cohort | 34 | 26.3 | 59.8 | 50.0 | 27.7 |  |  |  |  |  | 1 | 0 | 1 |
| Rogers et al. | 2021 | USA | MSA | RCT | 44 | 6.0 | 48.3 | 59.1 | 28.1 |  |  |  |  |  | 1 | 1 | 0 |
| Aiolfi et al. | 2022 | Italy | Toupet | Cohort | 71 | 12.0 | 67.1 | 21.1 | 26.5 |  |  |  |  |  | 0 | 1 | 0 |
| Riccardi et al. | 2022 | USA | MSA | Cohort | 336 | 12.0 |  |  |  |  |  |  |  |  | 0 | 1 | 0 |
| Snow et al. | 2022 | USA | TIF | Cohort | 49 | 10.5 | 54.4 | 37.0 | 27.5 |  |  |  |  |  | 1 | 1 | 0 |
| Wong et al. | 2022 | USA | LF | Cohort | 487 | 6.0 | 66.0 | 30.8 | 29.4 |  |  |  |  |  | 0 | 1 | 0 |
| Wong et al. | 2022 | USA | LF | Cohort | 487 | 12.0 | 66.0 | 30.8 | 29.4 |  |  |  |  |  | 0 | 1 | 0 |
| Wong et al. | 2022 | USA | LF | Cohort | 487 | 24.0 | 66.0 | 30.8 | 29.4 |  |  |  |  |  | 0 | 1 | 0 |
| Wong et al. | 2022 | USA | LF | Cohort | 487 | 60.0 | 66.0 | 30.8 | 29.4 |  |  |  |  |  | 0 | 1 | 0 |
| Asti et al. | 2023 | Italy | MSA | Cohort | 130 | 12.0 | 49.0 | 67.7 | 25.3 |  |  |  |  |  | 0 | 1 | 0 |
| Asti et al. | 2023 | Italy | Toupet | Cohort | 69 | 12.0 | 56.0 | 46.0 | 24.7 |  |  |  |  |  | 0 | 1 | 0 |
| Callahan et al. | 2023 | USA | MSA | Cohort | 46 | 1.0 | 56.0 | 45.7 | 28.7 |  |  |  |  |  | 0 | 1 | 0 |
| Callahan et al. | 2023 | USA | Nissen | Cohort | 356 | 1.0 | 65.0 | 27.8 | 29.6 |  |  |  |  |  | 0 | 1 | 0 |
| Callahan et al. | 2023 | USA | Toupet | Cohort | 207 | 1.0 | 67.0 | 35.7 | 29.0 |  |  |  |  |  | 0 | 1 | 0 |
| Callahan et al. | 2023 | USA | MSA | Cohort | 46 | 6.0 | 56.0 | 45.7 | 28.7 |  |  |  |  |  | 0 | 1 | 0 |
| Callahan et al. | 2023 | USA | Nissen | Cohort | 356 | 6.0 | 65.0 | 27.8 | 29.6 |  |  |  |  |  | 0 | 1 | 0 |
| Callahan et al. | 2023 | USA | Toupet | Cohort | 207 | 6.0 | 67.0 | 35.7 | 29.0 |  |  |  |  |  | 0 | 1 | 0 |
| **Author** | **Year** | **Country** | **Procedure** | **Design** | **N_Total** | **FollowUp** | **Age_Base** | **Male_Base** | **BMI_Base** | **EsoNone_Base** | **EsoA_Base** | **EsoB_Base** | **EsoC_Base** | **EsoD_Base** | **AET** | **Relief** | **DeMeester** |
| Callahan et al. | 2023 | USA | MSA | Cohort | 46 | 12.0 | 56.0 | 45.7 | 28.7 |  |  |  |  |  | 0 | 1 | 0 |
| Callahan et al. | 2023 | USA | Nissen | Cohort | 356 | 12.0 | 65.0 | 27.8 | 29.6 |  |  |  |  |  | 0 | 1 | 0 |
| Callahan et al. | 2023 | USA | Toupet | Cohort | 207 | 12.0 | 67.0 | 35.7 | 29.0 |  |  |  |  |  | 0 | 1 | 0 |
| Callahan et al. | 2023 | USA | MSA | Cohort | 46 | 24.0 | 56.0 | 45.7 | 28.7 |  |  |  |  |  | 0 | 1 | 0 |
| Callahan et al. | 2023 | USA | Nissen | Cohort | 356 | 24.0 | 65.0 | 27.8 | 29.6 |  |  |  |  |  | 0 | 1 | 0 |
| Callahan et al. | 2023 | USA | Toupet | Cohort | 207 | 24.0 | 67.0 | 35.7 | 29.0 |  |  |  |  |  | 0 | 1 | 0 |
| Callahan et al. | 2023 | USA | MSA | Cohort | 46 | 60.0 | 56.0 | 45.7 | 28.7 |  |  |  |  |  | 0 | 1 | 0 |
| Callahan et al. | 2023 | USA | Nissen | Cohort | 356 | 60.0 | 65.0 | 27.8 | 29.6 |  |  |  |  |  | 0 | 1 | 0 |
| Callahan et al. | 2023 | USA | Toupet | Cohort | 207 | 60.0 | 67.0 | 35.7 | 29.0 |  |  |  |  |  | 0 | 1 | 0 |
| Eriksson et al. | 2023 | USA | MSA | Cohort | 131 | 6.0 | 55.7 | 26.7 | 29.1 |  |  |  |  |  | 0 | 1 | 0 |
| Eriksson et al. | 2023 | USA | MSA | Cohort | 131 | 12.0 | 55.7 | 26.7 | 29.1 |  |  |  |  |  | 0 | 1 | 0 |
| Eriksson et al. | 2023 | USA | MSA | Cohort | 131 | 24.0 | 55.7 | 26.7 | 29.1 |  |  |  |  |  | 0 | 1 | 0 |
| Paranyak et al. | 2023 | Ukraine | Nissen | RCT | 60 | 6.0 | 51.3 | 38.3 | 27.8 |  |  |  |  |  | 0 | 1 | 0 |
| Paranyak et al. | 2023 | Ukraine | Toupet | RCT | 60 | 6.0 | 54.6 | 46.7 | 28.3 |  |  |  |  |  | 0 | 1 | 0 |
| Paranyak et al. | 2023 | Ukraine | Nissen | RCT | 60 | 12.0 | 51.3 | 38.3 | 27.8 |  |  |  |  |  | 0 | 1 | 0 |
| Paranyak et al. | 2023 | Ukraine | Toupet | RCT | 60 | 12.0 | 54.6 | 46.7 | 28.3 |  |  |  |  |  | 0 | 1 | 0 |
| Paranyak et al. | 2023 | Ukraine | Nissen | RCT | 60 | 24.0 | 51.3 | 38.3 | 27.8 |  |  |  |  |  | 0 | 1 | 0 |
| Paranyak et al. | 2023 | Ukraine | Toupet | RCT | 60 | 24.0 | 54.6 | 46.7 | 28.3 |  |  |  |  |  | 0 | 1 | 0 |
| Shen et al. | 2023 | China | TIF | Cohort | 16 | 6.0 | 48.9 | 84.6 | 24.0 | 23.1 | 46.1 | 23.1 | 7.7 | 0.0 | 1 | 1 | 1 |
| Shen et al. | 2023 | China | TIF | Cohort | 16 | 36.0 | 48.9 | 84.6 | 24.0 | 23.1 | 46.1 | 23.1 | 7.7 | 0.0 | 0 | 1 | 0 |
| Shen et al. | 2023 | China | TIF | Cohort | 16 | 60.0 | 48.9 | 84.6 | 24.0 | 23.1 | 46.1 | 23.1 | 7.7 | 0.0 | 0 | 1 | 0 |

*Note.* FollowUp is in months. For AET, Relief, and DeMeester score, a value of 1 indicates that the study measured that outcome, whereas a value of 0 indicates that the study did not measure that outcome.

**Table S6.** Jadad scores for randomized controlled trials

| Author | Randomization | Blinding | Withdrawals | Score |
| --- | --- | --- | --- | --- |
| Anvari et al. (2011) | 1 | 0 | 1 | 2 |
| Bell et al. (2019) | 1 | 0 | 1 | 2 |
| Cao et al. (2012) | 1 | 0 | 1 | 2 |
| Castelijns et al. (2018) | 1 | 0 | 1 | 2 |
| Chiu et al. (2017) | 1 | 0 | 0 | 1 |
| Djerf et al. (2016) | 1 | 0 | 1 | 2 |
| Fibbe et al. (2001) | 1 | 0 | 1 | 2 |
| Granderath et al. (2005) | 1 | 0 | 0 | 1 |
| Heikkinen et al. (2000) | 1 | 0 | 1 | 2 |
| Koch et al. (2011) | 1 | 0 | 1 | 2 |
| Koch et al. (2013) | 1 | 0 | 1 | 2 |
| Kosek et al. (2009) | 1 | 0 | 1 | 2 |
| Li et al. (2019) | 1 | 0 | 1 | 2 |
| Lundell et al. (2008) | 1 | 0 | 1 | 2 |
| Mahon et al. (2005) | 1 | 0 | 1 | 2 |
| Muller-Stich et al. (2015) | 1 | 0 | 1 | 2 |
| Ospanov et al. (2016) | 1 | 0 | 0 | 1 |
| Paranyak et al. (2023) | 1 | 0 | 1 | 2 |
| Qin et al. (2013) | 1 | 0 | 0 | 1 |
| Rinsma et al. (2015) | 1 | 0 | 0 | 1 |
| Rogers et al. (2021) | 1 | 0 | 0 | 1 |
| Shaw et al. (2010) | 1 | 0 | 1 | 2 |
| Trad et al. (2017) | 1 | 0 | 1 | 2 |
| Wang et al. (2015) | 1 | 0 | 1 | 2 |
| Witteman et al. (2015) | 1 | 0 | 1 | 2 |

**Table S7.** Newcastle-Ottawa scores for cohort designs

|  | Selection | | | |  | Comparability |  | Outcome | | |  |
| --- | --- | --- | --- | --- | --- | --- | --- | --- | --- | --- | --- |
| Author | Q1 | Q2 | Q3 | Q4 |  | Q1 |  | Q1 | Q2 | Q3 | Quality |
| Aiolfi et al. (2022) | * | * | * | * |  | * |  |  | * | * | Good |
| Allen et al. (1998) | * | * | * | * |  | * |  |  | * | * | Good |
| Aprea et al. (2012) | * | * | * | * |  | * |  |  | * | * | Good |
| Asti et al. (2023) | * | * | * | * |  | * |  |  | * | * | Good |
| Ayazi et al. (2020a) | * | * | * | * |  | * |  | * | * |  | Good |
| Balci et al. (2007) |  | * | * | * |  | * |  |  | * |  | Poor |
| Bell et al. (2012) | * | * | * | * |  | * |  | * | * |  | Good |
| Bell et al. (2014) | * | * | * | * |  | * |  |  | * | * | Good |
| Bonavina et al. (2021) | * | * | * | * |  | * |  |  | * |  | Poor |
| Brillantino et al. (2011) | * | * | * | * |  | * |  |  | * |  | Poor |
| Callahan et al. (2023) | * | * | * | * |  | * |  |  | * | * | Good |
| Demyttenaere et al. (2010) | * | * | * | * |  | * |  | * | * | * | Good |
| Eriksson et al. (2023) | * | * | * | * |  | * |  |  | * |  | Poor |
| Ferrari et al. (2020) | * | * | * | * |  | * |  | * | * |  | Good |
| Ferrari et al. (2021) | * | * | * | * |  | * |  | * | * |  | Good |
| Granderath et al. (2002) | * | * | * | * |  | * |  |  | * |  | Poor |
| He et al. (2021) |  | * | * | * |  | * |  | * | * |  | Good |
| Ihde et al. (2019) | * | * | * | * |  | * |  |  | * |  | Poor |
| Ilyashenko et al. (2018) |  | * | * | * |  | * |  | * | * |  | Good |
| Janu et al. (2019) | * | * | * | * |  | * |  |  | * |  | Poor |
| Kamolz et al. (2000) | * | * | * | * |  | * |  |  | * |  | Poor |
| Kamolz et al. (2001) | * | * | * | * |  | * |  |  | * |  | Poor |
| Kamolz et al. (2003) | * | * | * | * |  | * |  |  | * |  | Poor |
| Kothari et al. (2019) | * | * | * | * |  | * |  | * | * |  | Good |
| Lipham et al. (2012) | * | * | * | * |  | * |  |  | * |  | Poor |
| Lochegnies et al. (2001) |  | * | * | * |  | * |  |  | * | * | Good |
| Louie et al. (2019) | * | * | * | * |  | * |  | * | * | * | Good |
| Luketina et al. (2015) | * | * | * | * |  | * |  | * | * | * | Good |
| Muls et al. (2012) | * | * | * | * |  | * |  |  | * | * | Good |
| Neumayer et al. (2005) | * | * | * | * |  | * |  |  | * | * | Good |
| Ozmen et al. (2014) |  | * | * | * |  | * |  |  | * |  | Poor |
| Park et al. (2019) |  | * | * | * |  | * |  |  | * |  | Poor |
| Pidoto et al. (2006) |  | * | * | * |  | * |  | * | * |  | Good |
| Riccardi et al. (2022) | * | * | * | * |  | * |  |  | * |  | Poor |
| Richards et al. (2018) |  | * | * | * |  | * |  | * | * |  | Good |
| Roccato et al. (2021) |  | * | * | * |  | * |  | * | * |  | Good |
| Roy-Shapira et al. (2015) |  | * | * | * |  | * |  | * | * |  | Good |
| Saino et al. (2015) |  | * | * | * |  | * |  |  | * |  | Poor |
| Schneider et al. (2017) |  | * | * | * |  | * |  | * | * |  | Good |
| Shen et al. (2023) |  | * | * | * |  | * |  | * | * |  | Good |
|  | Selection | | | |  | Comparability |  | Outcome | | |  |
| Author | Q1 | Q2 | Q3 | Q4 |  | Q1 |  | Q1 | Q2 | Q3 | Quality |
| Slim et al. (2000) | * | * | * | * |  | * |  |  | * |  | Poor |
| Snow et al. (2022) |  | * | * | * |  | * |  | * | * | * | Good |
| Takeyama et al. (2004) | * | * | * | * |  | * |  |  | * | * | Good |
| Testoni et al. (2010) |  | * | * | * |  | * |  | * | * |  | Good |
| Testoni et al. (2015) | * | * | * | * |  | * |  | * | * | * | Good |
| Tezcaner et al. (2019) |  | * | * | * |  | * |  | * | * |  | Good |
| Turner et al. (2020) | * | * | * | * |  | * |  | * | * | * | Good |
| Wilson et al. (2014) | * | * | * | * |  | * |  |  | * | * | Good |
| Wong et al. (2022) | * | * | * | * |  | * |  | * | * |  | Good |
| Zehetner et al. (2006) | * | * | * | * |  | * |  | * | * |  | Good |
| Zugel et al. (2002) | * | * | * | * |  | * |  |  | * | * | Good |
